# Autoantibodies to the **β**_1_-Adrenoceptor from Patients with Periodontitis as a Risk Factor for Cardiac Dysfunction

**DOI:** 10.5402/2011/791393

**Published:** 2011-09-07

**Authors:** Marcela Segovia, Silvia Reina, Enri Borda, Leonor Sterin-Borda

**Affiliations:** ^1^Pharmacology Unit, School of Dentistry, University of Buenos Aires, 1122AAH Buenos Aires, Argentina; ^2^Argentine National Research Council (CONICET), Buenos Aires, Argentina

## Abstract

The presence of serum autoantibodies in periodontitis (P) patients against *β*_1_-adrenoceptor (*β*_1_-AR), using cardiac membranes or a synthetic *β*_1_-AR peptide corresponding to the second extracellular loop of human *β*_1_-AR as antigens, permit us to detect circulating antibody from 40 P patients but not in 20 normal individuals (control). Simultaneously, the P patients exhibited a decrease in HRV. Anti-*β*_1_-AR IgG titters correlated with the decrease in HRV of the same patients and the anti-*β*_1_-AR peptide IgG displayed partial agonist-like activity and modified the contractility of isolated atria, produced cyclic nucleotides, and inhibited the *β*_1_-AR agonistic activity of isoproterenol. We demonstrated in this study an association between periodontitis infection and an increased risk of cardiac disease, thereby highlighting the role of anti-*β*_1_-AR autoantibodies in alteration of myocardial contractility.

## 1. Introduction

Cardiovascular disease occurs as a result of a complex set of genetic and environmental factors [1]. Although the identity of the traditional risk factors has been established, they do not fully account for the risk of cardiovascular disease, suggesting that other mechanisms contribute to the pathogenic process [2]. Recently, several lines of evidence have implicated chronic inflammation in the etiology of cardiovascular disease [3].

Periodontitis is an inflammatory disease of tooth-supporting structures that leads to the destruction of connective tissue, loss of periodontal attachment, and resorption of alveolar bone [4].

The pathogenesis of the disease involves essential immunological factors associated with infections caused by bacteria into subgingival plaque [5]. Among the immunological factors, local synthesis of biological products (e.g., enzymes produced by cells of the supporting tissue) as well as bacterial-specific secretion of immunoglobulin (Ig), soluble inflammatory mediators, and/or cellular/tissue degradation products has been described [6–9]. It has been shown that endotoxins of plaque microorganisms penetrate gingival tissues and enter the bloodstream in sufficient numbers to cause a systemic lipopolysaccharide-specific antibody response [10].

However, in periodontal disease during the process of combating bacterial infection, the immune system may cause localized tissue damage and activate the systemic humoral immune response [11, 12]. Thus, the susceptibility to, and progression of, the disease is dependent upon not only the virulence of the microbial pathogens, but also the host response to regulation of tissue destruction.

Initial studies of autoimmunity in the pathogenesis of periodontal disease focused on detection of autoantibodies directed towards various self-antigens [13–15], such as autoantibodies against human gingival fibroblasts [16, 17]. Specifically, these antigingival fibroblast autoantibodies could interact with *β*_1_-ARs exhibiting adrenergic agonistic activity.

Moreover, autoantibodies with immune reactivity against *β*_1_-ARs of the myocardium have been described in the sera of patients with different cardiomyopathies associated with infection with parasites and viruses [18–22]. Increasing evidence indicates that periodontal disease is a risk factor for cardiovascular disease and stroke [23, 24]. The main underlying pathological pathway for cardiomyopathy associated with periodontal disease is atherosclerosis [2]. Increasing evidence suggests that the progression of atherosclerosis arises from a combination of dysfunction and inflammation of the endothelium. Inflammatory mediators derived from infected periodontal tissue or periodontopathic bacteria (or their products) could induce dysfunction of endothelial cells [2].

We demonstrated that serum autoantibodies from patients with periodontitis could react with the myocardial surface and with human *β*_1_-ARs. A correlation between the serum antibodies against myocardial *β*_1_-ARs and alteration in heart rate variability (HRV) could be determined. The capacity of *β*_1_-AR autoantibodies to modify atria contractility *in vitro* was also assessed.

## 2. Patients and Methods

### 2.1. Patients

The study group consisted of 40 male patients (mean age: 41 years; range: 32–50 years) with periodontitis attending periodontology clinics in the metropolitan area of Buenos Aires. Twenty healthy male subjects (mean age: 38 years; range: 30–46 years) were used as controls. The characteristic signs of periodontitis included the following: loss of clinical attachment; horizontal or/and angular alveolar bone loss; periodontal pocket formation; gingival inflammation. To be included in the study, at least six sites with ongoing periodontal disease were required. Measurements in patients with periodontitis included sites with alveolar bone loss >2 mm and pocket depth >5 mm with bleeding and attachment loss >3 mm. In healthy subjects (control group), the probing depth was <3 mm and attachment loss was <2 mm. None of the subjects (patients in group I and controls in group II) had systemic illnesses and they were not smokers. Patients had not received periodontal treatment or antibiotics within the preceding 5 months or anti-inflammatory drugs 3 weeks before the study. Also, it can be seen in Tables 1 and 2 the general characteristic of the study populations and selection index. All patients consented to participate in the study. The investigation was conducted according to the tenets of the Declaration of Helsinki.

### 2.2. HRV

The 20 patients with periodontitis and 20 normal subjects were studied according to a previous report [25]. Heart rate was recorded using an Oxford Medilog 2–24 miniature analog tape recorder (Oxford Instruments Medical Systems, Abingdon, UK). The recording speed was 2 mms^−1^. HRV was assessed from RR-interval files generated from 10 pm to 8 am analyzed in hourly epochs. The summary time-domain statistical measures of HRV calculated included the HRV long-term standard deviation of all NN (SDNN) indices. These indices represent variability over heart cycles and estimate high-frequency variation in the heart rate [25].

### 2.3. Human Sera and IgG Purification

Sera and the corresponding IgG were obtained from patients with periodontitis (group I) and from normal individuals (group II). Six milliliters of blood were obtained by venipuncture. Blood samples were allowed to clot at room temperature. The serum was separated by centrifugation at 2000 × g and stored at −20°C until used in assays. IgG was obtained by precipitation followed previous reports [17, 18].

### 2.4. Purification of Antipeptide Antibodies by Affinity Chromatography

The IgG fraction of group-I patients was independently subjected to affinity chromatography on the synthesized peptide covalently linked to Affi-Gel 15 (Bio-Rad, Richmond, CA, USA). The IgG fraction was loaded on the affinity column equilibrated with phosphate-buffered saline (PBS) according to previous reports [16, 17].

### 2.5. Membrane Preparation

Cardiac membranes from male rat Wistar strain were prepared as previously described [17, 18]. In brief, cells or atria were homogenized in an Ultra-Turrax homogenizer (IKA, Wilmington, NC, USA) at 4°C in 6 volumes of potassium phosphate buffer, 1 mM MgCI_2_, 0.25 M sucrose (pH 7.5) supplemented with 0.1 mM phenylmethyl sulphonyl fluoride (PMSF), 1 mM ethylenediamine tetra-acetic acid (EDTA), 5 *μ*g mL^−1^ leupeptin, 1 *μ*M bacitracin, and 1 *μ*M pepstatin A. The homogenate was centrifuged twice for 10 min at 3000 ×g, then at 10,000 ×g and 40,000 ×g at 4°C for 15 min and 90 min, respectively. The resulting pellets were suspended in 50 mM phosphate buffer fortified with the same protease inhibitors (pH 7.5).

### 2.6. ELISA Assay

Fifty micromililiters of peptide solution at 50 *μ*g/mL concentration or purified cardiac membranes (50 *μ*g/mL) in 0.1 M Na_2_CO_3_ buffer (pH 9.6) were used to coat microtiter plates at 4°C overnight. After blocking the wells with 2% bovine serum albumin in PBS for 1 h at 37°C, 10 *μ*L of a 1/30 dilution of sera of different concentrations or purified IgG from groups I and II were added in triplicate and allowed to react with the peptide for 2 h at 37°C. After thoroughly washing the wells with 0.05% Tween 20 in PBS, 100 *μ*L of 1 : 6000 goat anti-human IgG alkaline phosphate conjugate antibodies were added and incubated for 1 h at 37°C. After extensive washing, *p*-nitrophenylphosphate (1 mg/mL) was added as the substrate and the reaction stopped after 30 min. Optical density (OD) was measured at 405 nm with an ELISA reader. As a negative control, nonantigen paired wells with M_1_ cholinergic peptide and wells with no primary antiserum were also used. The results for each sample were expressed as the mean ± standard error of the mean (s.e.m.) of triplicate values.

### 2.7. Contractile Study

Male rats Wistar strain (200–250 g) were decapitated and atria quickly removed. Atria were placed in a glass chamber containing Krebs–Ringer bicarbonate (KRB) solution (pH 7.4) that was gassed with 5% CO_2_ in oxygen at 30°C. After a stabilization period of 30 min, spontaneous tension and frequency were recorded using a force transducer coupled to an ink-writing oscillograph, as previously described [18].

### 2.8. Assays for Cyclic Adenosine Monophosphate (cAMP) and Cyclic Guanosine Monophosphate (cGMP)

Rat atria (10 mg) were incubated in 1 mL KRB for 30 min and the *β*_1_ agonist isoproterenol (ISO) added in the last 15 min. When blockers were used, they were added 25 min before the addition of ISO. After incubation, atria were homogenized in 2 mL of absolute ethanol and centrifuged at 6000 ×g for 15 min at 4°C. Pellets were then rehomogenized in ethanol-water (2 : 1). Supernatants were collected and evaporated to dryness as described above. Cyclic AMP or cyclic GMP in the residue was dissolved in 400 *μ*L of 0.05 M sodium acetate buffer (pH 6.2). For determination of nucleotides, we used ELISA employing the protocol of production of cAMP and cGMP from Amersham Biosciences (Piscataway, NJ, USA). Results were expressed in pico moles per milligram of wet weight of tissue (pmol/mg tissue ww). 

### 2.9. Drugs

Stock solutions of ISO and atenolol were freshly prepared before each experiment. The *β*_1 _ peptide corresponded to the sequence of the second extracellular loop of the human *β*_1_-AR (HWWRA ESDEA RRCYN DPKCC DFVTN RC). An unrelated peptide derived from the second extracellular loop of the human M_2_ cholinoreceptor (VRTVE DGECY IQFFS NAAVT FGTA) was used as a control. Radioactive material, synthetic peptides, and *β*-adrenergic antagonists were from Dupont/New England Nuclear (Boston, MA, USA), Sigma Genosys (St. Louis, MO, USA), and Sigma-Aldrich (St. Louis, MO, USA), respectively.

### 2.10. Statistical Analysis

The Student's *t*-test for unpaired values was used to determine the levels of significance. Analysis of variance (ANOVA) and a *post hoc *test (Dunnett's method and Student-Newman-Keuls test) were employed when pairwise multiple comparison procedures were necessary. Differences between means were considered significant if *P* < 0.05.

## 3. Results

**Figure 1:**
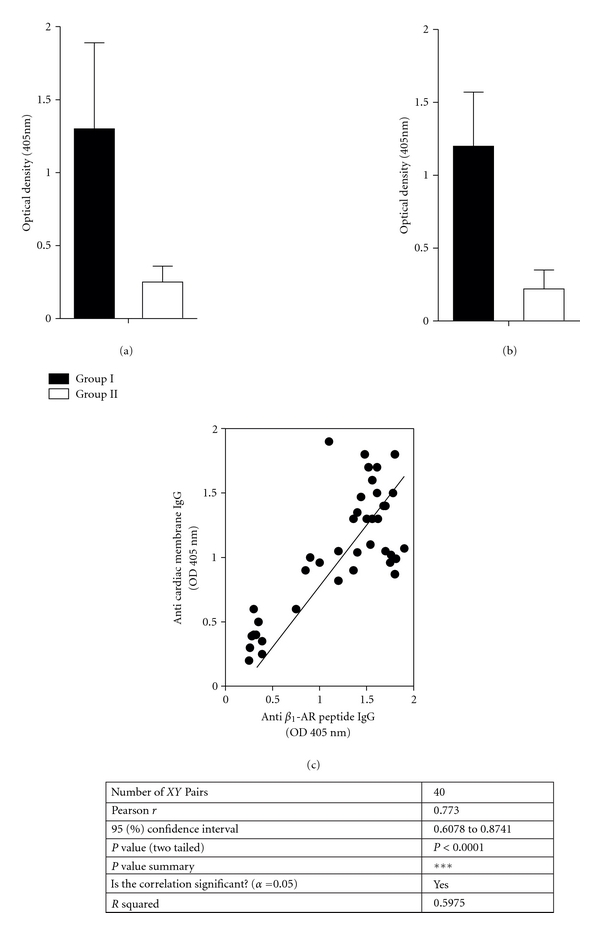
Immunoreactivity of circulating IgG antibodies against cardiac membrane (a) or against *β*_1_-AR synthetic peptide (b). The optical density (OD) for serum sample (1/30 dilution) from 40 periodontitis patients (group I) or 20 normal individuals (group II) were evaluated by duplicate. Values are mean ± SD. Cutoff values of OD 0.180 and 0.210 for cardiac membranes and anti-*β*_1_-AR synthetic peptide, respectively. Correlation (c) between titers of serum anti-*β*_1_-AR peptide IgG and anticardiac membrane IgG from periodontitis patients. Anti-*β*_1_-AR peptide IgG titters were plotted as function of anti cardiac membranes. The values correspond to 40 periodontitis patients.

ELISA assays were carried out to demonstrate if there was a correlation between the level of IgG in the serum of patients with periodontitis and cardiac membranes and the *β*_1_-AR.  Figure 1 shows the OD values for sera from patients with periodontitis (group I) and normal subjects (group II) using rat cardiac membrane (a) and *β*_1_-AR synthetic peptide (b) as coating antigens. The OD values obtained from group-I sera were always more than two standard deviations (>2 SD) from those of normal individuals.  Figure 1(c) shows a positive correlation between serum anticell cardiac membrane and anti-*β*_1_-AR peptide antibodies of the individual sera from group I. It was noted that 34/40 (85%) and 32/40 (80%) of patients' sera reacted positively against cardiac membranes and *β*_1_-AR synthetic peptide, respectively. When unrelated synthetic peptide (M_2_ cholinoceptor peptide) was used as a coating antigen, IgG from group I and from group II gave negative results (data not shown). 

**Figure 2:**
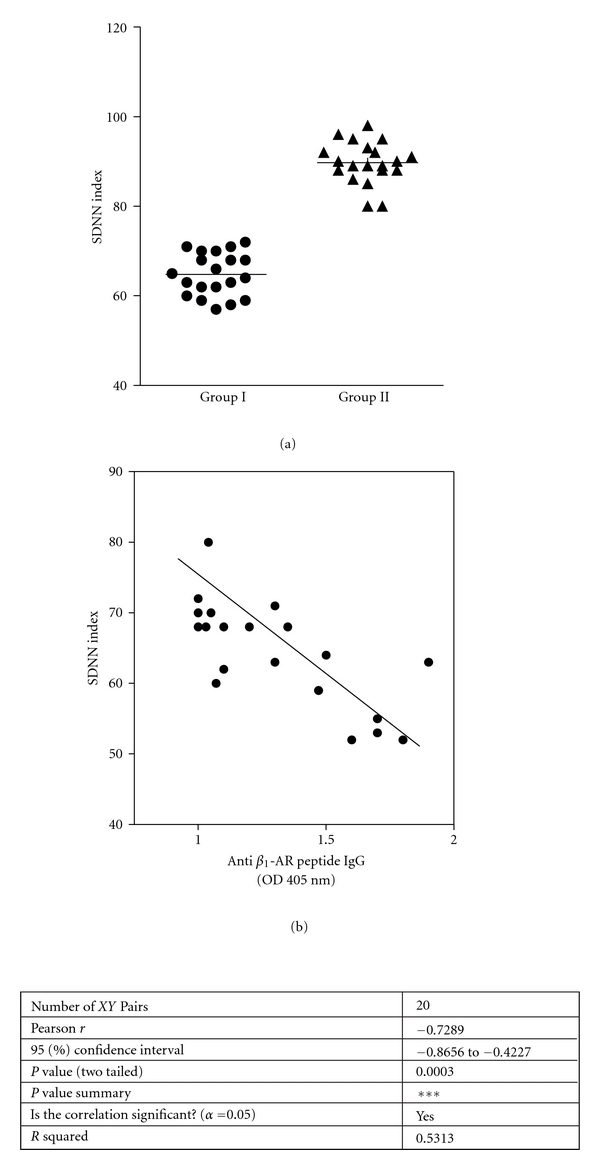
(a) Heart rate variability long-term standard deviation (SDNN) index in 20 patients with periodontitis (group I) and in 20 normal individuals (group II). (b) Correlation between values of the SDNN index and titers of serum anti-*β*_1_-AR peptide IgG. The SDNN index was plotted as a function of serum titers of anti-*β*_1_-AR peptide IgG corresponding to the same patients.

Knowing that the *β*_1_-AR is involved in regulating various cardiac parameters (including heart rate and HRV), we evaluated the HRV in 20 patients with periodontitis whose sera reacted positively against *β*_1_ synthetic peptide and in 20 normal individuals who acted as controls. The differences between patients with periodontitis and normal subjects with respect to the SDNN index are shown in Figure 2(a). There was a significant decrease in HRV (mean ± SD: 65.1 ± 9.2) in patients with periodontitis in comparison with the control group (mean ± SD: 90.3 ± 10). When the SDNN index was plotted as a function of serum titers, anti-*β*_1_-AR peptide IgG from each patient with periodontitis showed a negative correlation between serum anti-*β*_1_-AR peptide antibodies and the SDNN index (Figure 2(b)).

**Figure 3:**
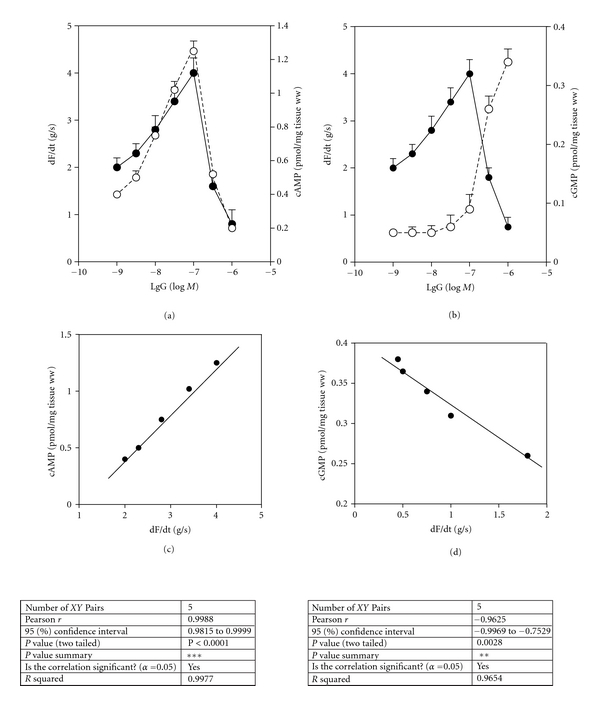
Effect of increasing concentrations of anti-*β*_1_-AR peptide IgG (IgG) on contractility (dF/dt) and production of cAMP and cGMP in isolated atria from rats. (a) Comparative effects of anti-*β*_1_-AR peptide IgG on dF/dt (•-•) and on cAMP production (○-○). (b) Comparative effects of anti-*β*_1_-AR peptide IgG on dF/dt (•-•) and on cGMP production (○-○). (c) Positive correlation between cAMP and dF/dt (corresponding data to A values). (d) Negative correlation between cGMP and dF/dt (corresponding data to B values). Values are mean ± s.e.m. of 20 patients with periodontitis.

We observed that patients with periodontitis showed a profound alteration in cardiac contractility *in vivo*, so we wanted to ascertain if serum anti-*β*_1_-AR antibodies could trigger cardiac *β*_1_-AR functional alterations in atria. For this purpose, we selected the 20 patients with periodontitis who showed a decrease in HRV and whose sera were positive against *β*_1_-adrenergic synthetic peptide in the ELISA assay. The IgG fraction was independently subjected to affinity chromatography and eluted from the column covalently linked to *β*_1_-adrenergic synthetic peptide (anti-*β*_1_-AR peptide IgG). Increasing concentrations of anti-*β*_1_-AR peptide IgG were applied to isolated spontaneously beating rat atria and changes in the magnitude of contractility (dF/dt) measured (Figure 3). 

Anti-*β*_1_-AR peptide IgG behaved as a partial *β*_1_-adrenergic agonist; increasing dF/dt at low concentrations and at higher concentrations, autoantibodies decreased dF/dt (Figures 3(a) and 3(b)). The stimulatory and inhibitory effects were accompanied by modification of cAMP production (Figure 3(a)) and cGMP production (Figure 3(b)). cAMP production correlated positively with the stimulatory effect of the antibodies (Figure 3(c)), but cGMP production correlated negatively with the inhibitory effect of the antibodies (Figure 3(d)).  Table 3 shows the effect of blockade upon the effect of IgG on dF/dt, cAMP and cGMP. For the *β*_1_-AR, the stimulatory and inhibitory effects were almost abolished by pretreatment of atria with 1  × 10^−7^ M atenolol (*β*_1_-AR antagonist) and by 1 × 10^−5^ M *β*_1_-adrenergic synthetic peptide. The IgG from normal subjects and the nonpeptide fraction gave negative results (data not shown). 

To ascertain if anti-*β*_1_-AR peptide IgG interacted with the same cardiac *β*_1_-AR epitope as an authentic agonist (ISO), we studied the action of anti-*β*_1_-AR peptide antibody upon ISO. The maximal positive effects of 5 × 10^−8^ M ISO on dF/dt (g/s: 5.3 ± 0.5, *n* = 5) and the cAMP stimulation (pmol/mg tissue ww: 2.8 ± 0.3, *n* = 5) were blunted by 5 ×10^−7^ M anti-*β*_1_-AR peptide IgG.

## 4. Discusion

We previously demonstrated that patients with periodontitis have functional serum IgG that interacts with the *β*_1_-AR of gingival fibroblasts. In the present study, we confirmed the presence of anti-*β*_1_-AR autoantibodies in the sera of patients with periodontitis and provided evidence that these sera can also recognize the cardiac membranes of rats. Most of the sera that reacted positively against rat cardiac membranes showed positive immune reactivity to human *β*_1_-AR synthetic peptide. Furthermore, a significant correlation between anticardiac membrane antibodies and anti-*β*_1_-AR peptide IgG was observed.

Using a synthetic peptide with an identical amino acid sequence of the second extracellular loop of human *β*_1_-AR, we established that the *β*_1_-AR is a target for the anticardiac autoantibodies described in patients with periodontitis. Due to the strong homology between rodent and human *β*_1_-AR peptides [26], one can assume that this is the one recognized by the autoantibodies. It has been shown that the second extracellular loop of cardiovascular G-protein-coupled receptors is an antigenic target for the generation of autoantibodies in patients with cardiomyopathy [27]. Therefore, we studied the *β*_1_-AR-mediated effect of autoantibodies on isolated rat atria using affinity-purified anti-*β*_1_-AR peptide IgG from patients with periodontitis. 

HRV is a powerful predictor of cardiovascular mortality in healthy people, those who have had a myocardial infarction, and in patients with heart failure. An elevated resting heart rate, reduced HRV, or both are negative prognostic factors independent of other clinical parameters, including left ventricular function [28]. Impaired control of HRV is linked to a deleterious sympathovagal imbalance characterized by predominance of the sympathetic nervous system [28].

In the present study, the finding of a correlation between decrease in HRV and the presence of serum anticardiac membrane autoantibodies and anti-*β*_1_-AR autoantibodies in patients with periodontitis allows us to suggest that the lower HRV observed in such patients is due to fixation of antibodies upon receptors. HRV alteration may be a consequence of activation of the *β*_1_-AR induced by antibody *β*_1_-AR interaction on the cardiac sarcolemma. 

We also provide evidence that isolated fractions of IgG enriched in anti-*β*_1_-AR peptide antibody could display partial agonistic activity on contractility in isolated atria. The partial agonistic activity of autoantibodies observed *in vitro* mimics the action on HRV of the partial *β*_1_-adrenergic agonist celiprolol described *in vivo*. However, the combination of celiprolol with atenolol did not prevent the fall in HRV triggered by the partial agonist [25]. Hence, the antibody-mediated partial *β*_1_ agonistic activity could be responsible for the decrease in HRV in patients with periodontitis.

The increase in contractility observed at low concentrations of IgG correlated with the increase in production of cAMP, whereas the decrease in contractility triggered by higher concentrations of IgG correlated with an increase in cGMP production. The increase and decrease in contractility are related to *β*_1_-AR function because they were blunted by atenolol. The increase in cGMP production which could limit the increase in contractility of *β*-adrenergic stimulation of isolated rat atria has been established [29]. 

The interaction of anti-*β*_1_-AR autoantibodies with the receptor has two functional implications: (i) it directly modifies the sympathetic activity of the myocardium; (ii) it decreases the effectiveness of the authentic agonist ISO. This raises the question: could there be a common explanation for the abnormal sympathetic activity in the cardiovascular system mediated by cardiac *β*_1_-AR autoantibodies? If so, antibody fixation could increase sympathetic activity for an extended period and result in a chronically elevated heart rate. Simultaneously, the antagonistic activity of the autoantibodies could lead to deleterious sympathovagal imbalance, resulting in functional deregulation associated with pathologic remodelling, myocyte apoptosis, and alteration of calcium handling that leads to myocardial ischemia, a decrease in contractile function, and an increased risk of ventricular arrhythmias [30].

## Figures and Tables

**Table 1:** Characteristic of the study populations.

Demography and risk factors	Periodontitis patients group I (*n* = 40)	Healthy subjects group II (*n* = 20)
Gender		
Male	36	18
Female	4	2
Education level		
Elementary school	38	18
High school	2	2

BMI (range kg/m^2^)	From 20 to 25	From 19 to 22

Measure blood pressure (mmHg)		
Measure SBP (mean ± SD)	138 ± 20	118 ± 14
Measure DBP (mean ± SD)	87 ± 12	75 ± 10

Laboratory examination		
Cholesterol total (mean ± SD), mg/dl	170 ± 21	176 ± 22
LDL (mean ± SD), mg/dl	129 ± 14	130 ± 21
HDL (mean ± SD), mg/dl	34 ± 10	32 ± 11

BMI: Body Mass Index (range ≥27 kg/m^2^); SBP: Systolic Blood Pressure. DBP: Diastolic Blood Pressure; LDL: Low-density lipoprotein; HDL: High-density lipoprotein.

**Table 2:** Periodontitis selection index.

Parameters	Numerical ranges
PPD	≥6 mm
CAL	≥6 mm

PPD: pocket probing depth; CAL: clinical attachment loss.

**Table 3:** Influence of *β*_1_ blockade on the stimulatory and inhibitory effects of anti-*β*_1_-AR peptide IgG.

Addition	dF/dt	cAMP	cGMP
Basal	1.8 ± 0.21	0.38 ± 0.03	0.041 ± 0.004
*β*_1_-AR IgG (5 × 10^−8^ M)	3.9 ± 0.32*	1.25 ± 0.11*	0.051 ± 0.004
*β*_1_-AR IgG (5 × 10^−8^ M) + atenolol	2.1 ± 0.19**	0.41 ± 0.04**	—
*β*_1_-AR IgG + *β*_1_-AR peptide	2.3 ± 0.23**	0.42 ± 0.03**	—
Normal IgG (5 ×10^−8^ M)	1.9 ± 0.18	0.40 ± 0.02	0.039 ± 0.04

Basal	2.1 ± 0.22	0.40 ± 0.03	0.042 ± 0.004
*β*_1_-AR IgG (5 ×10^−7^ M)	0.6 ± 0.05*	0.21 ± 0.02*	0.350 ± 0.03*
*β*_1_-AR IgG (5 ×10^−7^ M) + atenolol	1.7 ± 0.15**	0.38 ± 0.03**	0.122 ± 0.02**
*β*_1_-AR IgG + *β*_1_-AR peptide	1.9 ± 0.20**	0.37 ± 0.02**	0.101 ± 0.02**
Normal IgG (5 ×10^−7^ M)	2.0 ± 0.19	0.42 ± 0.03	0.043 ± 0.003

Values are mean ± s.e.m. of ten patients with periodontitis in each group carried out in duplicate. Contractility (dF/dt: g/s), and levels of cAMP (pmol/mg tissue ww) and cGMP (pmol/mg tissue ww) were measured after incubation for 15 min with rat atria in the presence of anti-*β*_1_-AR peptide IgG (*β*_1_-AR IgG) and normal IgG or in the absence (basal) of IgG. Atenolol (5×10^−7^ M) and *β*_1_-AR peptide (5×10^−5^ M) were added before the IgG. *P < 0.001 versus basal; **P < 0.001 versus *β*_1_-AR IgG alone.
